# Estimating recruitment rates for routine use of patient reported outcome measures and the impact on provider comparisons

**DOI:** 10.1186/1472-6963-14-66

**Published:** 2014-02-11

**Authors:** Andrew Hutchings, Jenny Neuburger, Jan van der Meulen, Nick Black

**Affiliations:** 1 Department of Health Services Research & Policy, London School of Hygiene & Tropical Medicine, 15-17 Tavistock Place, London WC1H 9SH, UK

**Keywords:** Patient reported outcome measures, Recruitment rates, Data linkage

## Abstract

**Background:**

The routine use of patient reported outcome measures (PROMs) aims to compare providers as regards the clinical need of their patients and their outcome. Simple methods of estimating recruitment rates based on aggregated data may be inaccurate. Our objectives were to: use patient-level linked data to evaluate these estimates; produce revised estimates of national and providers’ recruitment rates; and explore whether or not recruitment bias exists.

**Methods:**

Case study based on patients who were eligible to participate in the English National PROMs Programme for elective surgery (hip and knee replacement, groin hernia repair, varicose vein surgery) using data from pre-operative questionnaires and Hospital Episode Statistics. Data were linked to determine: the eligibility for including operations; eligibility of date of surgery; duplicate questionnaires; cancelled operations; correct assignment to provider. Influence of patient characteristics on recruitment rates were investigated.

**Results:**

National recruitment rates based on aggregated data over-estimated the true rate because of the inclusion of ineligible operations (from 1.9% - 7.0% depending on operation) and operations being cancelled (1.9% - 3.6%). Estimates of national recruitment rates using inclusion criteria based on patient-level linked data were lower than those based on simple methods (eg hip replacement was 73% rather than 78%).

Estimates of provider’s recruitment rates based on aggregated data were also adversely affected by attributing patients to the wrong provider (2.4% - 4.9%). Use of linked data eliminated all estimates of over 100% recruitment, though providers still showed a wide range of rates.

While the principal determinant of recruitment rates was the provider, some patients’ socio-demographic characteristics had an influence on non-recruitment: non-white (Adjusted Odds Ratio 1.25-1.67, depending on operation); most deprived socio-economic group (OR 1.11-1.23); aged over 75 years (OR 1.28-1.79). However, there was no statistically significant association between providers’ recruitment rates and patients’ pre-operative clinical need.

**Conclusions:**

Accurate recruitment rates require the use of linked data to establish consistent inclusion criteria for numerators and denominators. Non-recruitment will bias comparisons of providers’ pre-operative case-mix and may bias comparisons of outcomes if unmeasured confounders are not evenly distributed between providers. It is important, therefore, to strive for high recruitment rates.

## Background

There is increasing interest in using patient reported outcome measures (PROMs) to assess the quality of providers of surgery, most notably in Sweden [1] and, more recently, in England [2]. The National PROMs Programme that started in England in April 2009 covered four common operations and aimed to allow comparisons of providers in two principal ways: the appropriateness of patients undergoing surgery according to their preoperative health status and quality of life; and the outcome of care as judged by the change in health status and quality of life [3].

One particular concern is the extent to which incomplete recruitment of patients might bias comparisons of providers. This raises two methodological questions: are the estimates of recruitment rates accurate; and to what extent do those recruited differ from those not recruited?

As regards the first question, simple recruitment rates for each provider are routinely reported by the Health and Social Care Information Centre (HSCIC) [4] based on the number of completed pre-operative PROM questionnaires and the number of procedures undertaken according to a routine administrative data source, the Hospital Episode Statistics (HES). The shortcomings of this approach are evident in the number of providers who are reported as having more than 100% recruitment.

Potential causes of inaccuracies in the estimates of the numerator include: patients being included who underwent an ineligible procedure; despite having completed a pre-operative questionnaire, the procedure did not take place; the procedure was delayed such that it took place outside the time period being assessed; and patients being inadvertently asked to complete more than one pre-operative questionnaire, creating duplicate entries. Errors in the denominator can arise because patients are attributed to the wrong provider, a particular risk when operations are sub-contracted to another provider. To investigate the extent to which these problems occur, data on individual patients completing a pre-operative questionnaire need to be linked to their data in the routine administrative data. In this way it is possible to ensure the individual patients included in the numerator are the same individuals as in the denominator.

Even having corrected for such factors, recruitment rates for providers are likely to vary. And if recruitment is associated with patient characteristics that are, in turn, associated with the probability of the level of benefit patients will gain from the intervention, then comparisons of providers’ outcomes may be confounded. Given that the case-mix of providers is known to differ, the impact that any specific characteristic (eg age) has will also differ, reducing the external validity of comparisons of providers [5]. It is therefore important to identify patient characteristics associated with non-recruitment.

Given these concerns about simple methods of estimating recruitment rates based on aggregated data, our three objectives were to: use patient-level linked data to evaluate these estimates; produce revised estimates of national and providers’ recruitment rates; and explore whether or not recruitment bias exists.

## Methods

Analysis of secondary data from the routine use of PROMs in the English NHS for patients undergoing hip replacement, knee replacement, groin hernia repair, and varicose vein (VV) surgery were used to investigate the objectives. These procedures had been selected for the programme by the Department of Health for England as commonly occurring elective operations.

### Eligible procedures

All elective patients funded by the NHS in England were eligible for inclusion if they underwent one of the following procedures: for hip surgery, unilateral, primary or revision surgery; total hip replacement, femoral head prosthesis only, acetabular cap prosthesis only. For knee surgery, total knee replacement or unicondylar surgery, unilateral, primary or revision. Groin hernia repairs included operations for inguinal, femoral and incisional hernias, unilateral, open or laparoscopic, primary or recurrent. For VV surgery, all modalities were eligible for inclusion: surgery, radiofrequency ablation, endovenous laser therapy, endoscopic perforator surgery, injection or foam sclerotherapy.

### Data sources

Two sources of data on patients undergoing surgery between October 2009 and September 2010 were used: pre-operative PROMs data for all patients who completed a questionnaire; and HES data on all patients whose episode included the relevant surgical procedures (based on 3-character OPCS codes). We received permission from the NHS Information Centre for use of the data. Analyses were restricted to NHS Trusts with at least 50 eligible HES episodes. Ethics committee approval was not necessary as this was secondary analysis of existing databases.

Linking patients who completed a pre-operative PROMs questionnaire to HES depended on the HSCIC having successfully attaching a HES_ID to each questionnaire. HES_IDs are patient identifiers based on the patient’s NHS Number and other identifiers. If the HSCIC is unable to identify a patient who completed a PROMs questionnaire in HES, they are not able to generate and attach a HESID.

### Establishing accurate linked data

Step 1: Eliminating duplicate pre-operative questionnaires

HES data and pre-operative questionnaire data were used to check if a patient had inadvertently completed a questionnaire twice. The questionnaire with the best link to a hospital episode (by date and provider) was retained.

Step 2: Criteria for handling pre-operative questionnaires linked to more than one hospital episode

If links to more than one hospital episode were found, the one that most closely fitted in clinical terms and closest agreement of dates was used. Requirements for linkage were fairly unrestricted to maximise the success rate. Links were made even if:

• the provider identified in HES was different to the one that supplied the questionnaire.

• the date of surgery in HES was within 30 days before or 180 days after the date the questionnaire was completed.

• the date of surgery recorded in the post-operative PROM questionnaire agreed with the date in HES.

Step 3: Identifying patients whose operation was cancelled

The PROMs database included a variable which indicates the stage the patient is at. This includes the option of ‘operation cancelled’.

Step 4: Excluding ineligible patients

If having linked the pre-operative questionnaires to a hospital episode, it was apparent the procedure was not eligible for inclusion, it was excluded. This arose most frequently when patients underwent resurfacing of their hip or knee joint (rather than joint replacement) and for those undergoing an incisional hernia repair in which the anatomical site was not stated. Patients were also excluded if the linked hospital episode fell outside the study period (October 2009-September 2010) even if the questionnare fell within it.

Step 5: Assigning eligibility to patients with unlinked pre-operative questionnaires

Having undertaken these steps, there remained some patients who had completed a questionnaire but could not be linked to HES (either because they had no HESID or no hospital episode could be found). As the eligibility of these questionnaires was uncertain, eligibility was assigned according to the proportion of eligible questionnaires found among patients who had been linked.

### Investigating associations between patient characteristics and recruitment

Four socio-demographic characteristics of patients were available from HES data: age, sex, ethnicity, and deprivation measured using the Index of Multiple Deprivation (IMD) [6]. The characteristics of patients for whom eligible hospital episodes were matched to a questionnaire were compared with those not matched to a questionnaire (taken as a proxy for non-recruitment). Multivariable logistic regression was used to examine the association between the characteristics and recruitment for each procedure. Providers were included as random effects (intercepts) to allow for differences in recruitment rates between providers.

While that approach is inevitably limited to those four patient characteristics, it is possible to investigate other factors indirectly by considering whether providers’ recruitment rates are associated with the health status of their patients. We explored the correlation between recruitment rates and mean disease-specific PROM scores (eg Oxford Hip Score) and mean EQ-5D scores for each of the operations. The impact of a 10% higher recruitment rate on the mean scores for each operation was also calculated.

## Results

### Establishing accurate linked data

It was possible to establish a database that linked the majority of pre-operative questionnaires. The total numbers of patients for each procedure according to the HES data are shown in the first row of Table 1 (eg 66 598 hip replacements). The number of completed pre-operative questionnaires received by the Information Centre is shown in row 2 (eg 52 183 for hip replacement). These two sets of data are the basis of the ‘official’ national recruitment rates (eg 52 183/66 598 = 78.4%).

**Table 1:** Number (%) of patients in HES who completed pre-operative questionnaires who were ineligible, of uncertain eligibility and were eligible for inclusion; and impact on estimated recruitment rate

	**Hip replacements**	**Knee replacements**	**Groin hernia repair**	**VV surgery**
**No patients in HES**	66 598	74 827	68 886	34 909
**No patients who completed pre-operative questionnaires**	52 183	60 621	37 713	15 588
No (%) of patients who completed questionnaires of **uncertain eligibility**	6 231 (11.9)	7 366 (12.2)	7 161 (19.0)	1 530 ( 9.8)
* No hospital episode*	*4 771 (9.1)*	*5 704 (9.4)*	*6 006 (15.9)*	*1 085 (7.0)*
* No HES_ID*	*1 460 (2.8)*	*1 662 (2.8)*	*1 155 (3.1)*	*445 (2.8)*
No (%) of patients who completed questionnaires who were **ineligible**	3 472 ( 6.7)	6 466 (10.7)	2 041 ( 5.4)	682 ( 4.4)
* Operation cancelled*	*1 236 (2.4)*	*2 185 (3.6)*	*786 (2.1)*	*302 (1.9)*
* Duplicate questionnaire*	*185 (0.4)*	*65 (0.1)*	*139 (0.4)*	*96 (0.6)*
* Ineligible procedure*	*2051 (3.9)*	*4216 (7.0)*	*1116 (3.0)*	*284 (1.9)*
No (%) of patients who completed questionnaires who were **eligible**	42 480 (81.4)	46 879 (77.3)	28 515 (75.6)	13 376 (85.8)
**National recruitment proportion:**				
Official (No of completed questionnaires/No patients in HES)	78.4	81.0	54.7	44.7
Best estimate (excluding ineligible)	73.1	72.4	51.8	42.7
Conservative estimate (excluding ineligible & uncertain eligible)	63.8	62.6	41.4	38.3

#### Patients who completed questionnaires but of uncertain eligibility

If questionnaires could not be linked to a HES episode, their eligibility for inclusion (ie they underwent the procedures being studied) was uncertain. The proportion that could not be linked varied from 9.8% to 19.0% by procedure (shown in row 3). For hip replacement this was true for 6 231 (11.9% of completed questionnaires). Our inability to link these patients arose mostly because no hospital episode could be found in HES and sometimes because they had no HES_ID.

Among those successfully linked, several reasons emerged as to why estimates of recruitment rates based on a simple comparison of numbers of questionnaires and hospital episodes rather than on linked data will be inaccurate.

#### Patients who completed questionnaires but were ineligible

There were three main reasons why patients were ineligible. First, having completed a questionnaire their operation was cancelled or postponed. This varied from 1.9% for VV surgery to 3.6% for knee replacement (Table 1). Second, some patients inadvertently completed two questionnaires though this occurred rarely, making up only 0.1-0.6% of all cases.

Third, some patients underwent operations that were not eligible for inclusion. This varied from 1.9% in VV surgery to 7.0% in knee surgery. For the two orthopaedic operations, this most frequently arose for patients undergoing resurfacing operations (75 hips, 3086 knees), bilateral operations (192 hips, 441 knees), non-elective operations (157 hips, 69 knees), non-NHS patients (247 hips, 159 knees) and the operation not being the initial reason they were admitted to hospital (204 hips, 206 knees).

Most (865) of the 1116 ineligible hernia operations were repairs of incisional hernias in which the site was not clearly coded as the groin. It is likely that these patients were in fact eligible but without the confirmatory site code, they couldn’t be included. Other reasons for ineligibility were 75 non-elective admissions, 56 non-NHS patients, and 113 who were initially admitted for another reason. Ineligibility of VV surgery patients was mostly due to the lack of a diagnostic code confirming the operation was carried out for VVs. These operations were probably eligible but, to be consistent with the denominator calculation, were excluded from the estimation of recruitment rate.

#### Ensuring operation within study period

The number of patients that had to be excluded for being outside the time period was balanced by a similar number who had been missed and had to be added (therefore not shown in Table 1). The numbers involved represented about 2% of all cases for hip and knee replacements and 1% for the other operations. The lower proportion for hernia repair and VV surgery was probably because more of those patients completed questionnaires on the day of surgery (rather than at pre-operative assessment clinics) thus minimising this particular risk.

#### Ensuring attribution to correct provider

While not affecting the national recruitment rate, a few eligible linked questionnaires (between 2.4% for groin hernia repair and 4.9% for knee replacements) were attributed to a different provider in HES than that indicated on their questionnaire. This was corrected when estimating providers’ rates.

### Estimating recruitment rates

After removing pre-operative questionnaires that were ineligible, the national recruitment rate inevitably fell (indicated as Best estimate in Table 1). For hip replacements the rate (based simply on the number who completed a questionnaire divided by the number recorded in HES) fell from 78.4% to 73.1%; for knee replacement from 81.0 to 72.4%, for hernia repair from 54.7 to 51.8%; and for VV surgery from 44.7 to 42.7%.

If a very conservative approach were to be adopted and all patients of uncertain eligibility were assumed to be ineligible and were excluded, the recruitment rate would be even lower (eg 63.8% for hip replacement).

**Figure 1:**
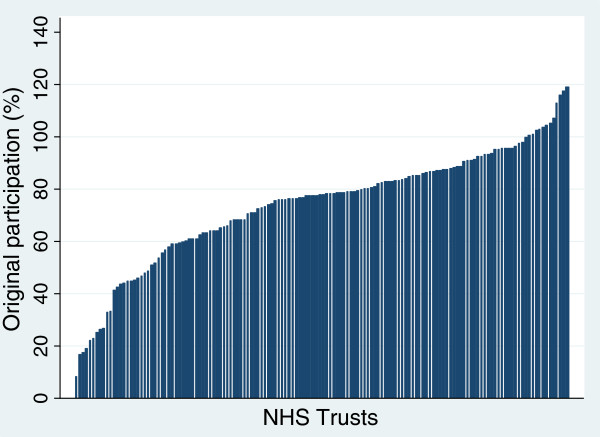
Hip replacements: original recruitment rates for NHS Trusts (n = 144).

**Figure 2:**
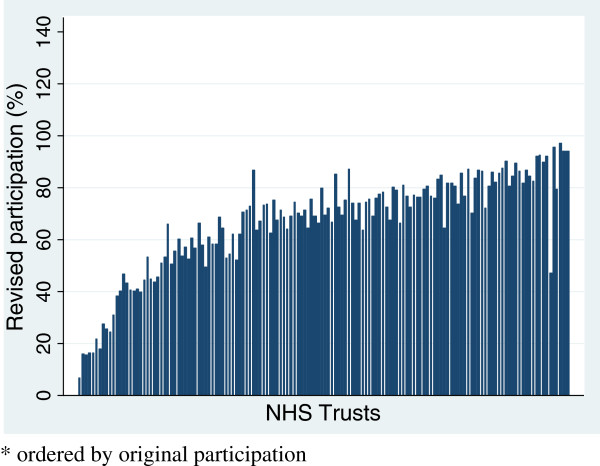
**Hip replacements: revised recruitment rates for NHS Trusts (n = 144).** * ordered by original participation.

**Figure 3:**
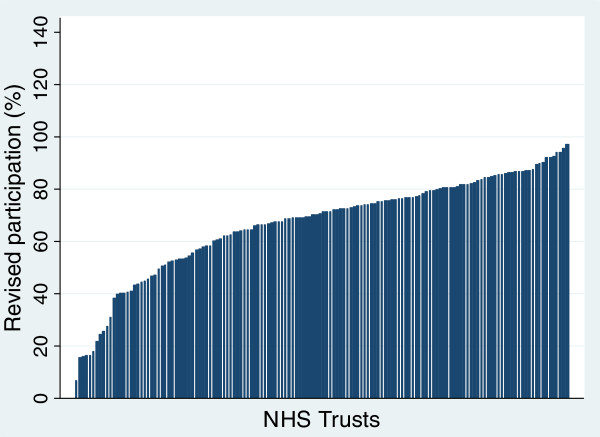
Hip replacements: revised recruitment rate for NHS Trusts (n = 144).

Figure 1 shows recruitment rates for hip replacement for 144 providers based on the initial simple approach. Twelve providers had recruitment rates above 100%. Figure 2 shows that after using linked data all providers were below 100%. For most there was only a small change but for a few it was substantial. Figure 3 shows the revised distribution revealing that while a quarter of providers recruited at least 80% of eligible patients a fifth managed less than 50%. The pattern for knee replacements was similar to that of hip replacements (Additional file 1). For groin hernia repair, for which no providers had estimates above 100% in the original estimates, with linked data over half the providers were found to have recruited less than 50% of patients. For VV surgery, almost two-thirds of providers had recruitment rates below 50% although a couple managed to recruit over 90%.

### Identifying characteristics of patients not recruited

Rates of non-recruitment for each operation by socio-demographic characteristics of patients is shown in Table 2 together with adjusted odds ratios for non-recruitment. There was little difference in recruitment by sex except for groin hernia repair in which women were less likely to be recruited (OR 1.52). Association with age varied by operation: recruitment became less likely with increasing age in VV surgery whereas age only appeared to have an influence in hip replacement and hernia repair in the oldest group (OR 1.28 and 1.37 respectively) and had little influence in knee replacement.

**Table 2:** Proportion of non-recruited patients by socio-demographic characteristics and adjusted odds ratio (OR) for non-recruitment

	**Procedure (number not recruited/total number in HES)**							
	**Hip replacement**		**Knee replacement**		**Groin hernia repair**		**VV surgery**	
	**(24 071/66 598)**		**(27 982/74 827)**		**(40 418/68 886)**		**(21 508/34 909)**	
	**%**	**OR (95% CI)**	**%**	**OR (95% CI)**	**%**	**OR (95% CI)**	**%**	**OR (95% CI)**
**Sex**								
Male	35.6	Ref	37.0	Ref	57.7	Ref	62.7	Ref
Female	36.6	1.04 (1.00, 1.08)	37.8	1.03 (0.99, 1.06)	67.0	1.52 (1.43, 1.61)	61.0	0.99 (0.94, 1.04)
**Age (years)**								
Under 55	36.5	Ref	39.8	Ref	57.8	Ref	59.3	Ref
55-64	33.4	0.92 (0.87, 0.99)	35.8	0.85 (0.79, 0.91)	55.7	0.95 (0.91, 0.99)	62.6	1.14 (1.08, 1.22)
64-75	34.5	0.99 (0.93, 1.05)	36.4	0.88 (0.82, 0.94)	57.8	1.05 (1.01, 1.10)	65.1	1.26 (1.17, 1.36)
75 and over	39.7	1.28 (1.21, 1.36)	39.4	1.03 (0.97, 1.11)	63.7	1.37 (1.31, 1.44)	72.4	1.79 (1.61, 1.99)
**Ethnicity**								
White	36.0	Ref	36.9	Ref	57.4	Ref	61.4	Ref
Non-white	46.7	1.49 (1.30, 1.70)	48.1	1.67 (1.55, 1.80)	68.8	1.44 (1.32, 1.56)	70.8	1.25 (1.12, 1.39)
Not known	36.7	1.01 (0.95, 1.08)	37.3	0.98 (0.92, 1.04)	60.9	1.01 (0.96, 1.07)	59.3	1.03 (0.95, 1.11)
**Deprivation (IMD quintile)**								
Least deprived	35.0	Ref	36.7	Ref	56.4	Ref	62.4	Ref
4th	35.1	1.03 (0.98, 1.08)	36.4	1.01 (0.96, 1.06)	57.8	1.04 (0.99, 1.09)	61.0	0.96 (0.88, 1.03)
3rd	36.1	1.08 (1.03, 1.14)	37.0	1.04 (0.99, 1.09)	58.3	1.05 (1.00, 1.11)	61.4	1.00 (0.92, 1.08)
2nd	37.9	1.17 (1.11, 1.24)	38.6	1.09 (1.04, 1.15)	59.4	1.08 (1.02, 1.14)	61.4	0.98 (0.90, 1.06)
Most deprived	38.5	1.20 (1.13, 1.28)	39.5	1.17 (1.10, 1.24)	61.5	1.23 (1.15, 1.30)	62.4	1.11 (1.02, 1.21)

**Figure 4:**
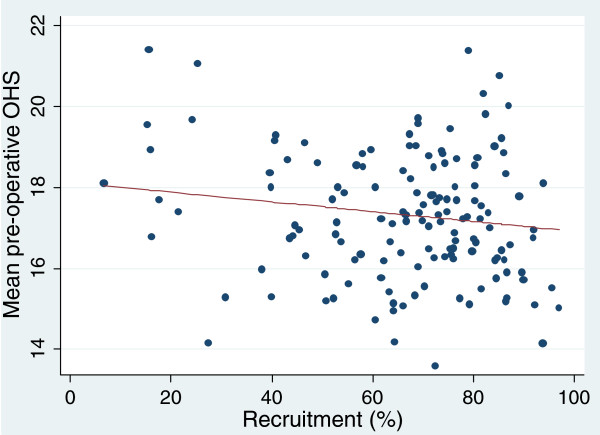
Association between providers’ recruitment rates and mean Oxford Hip Score (correlation - 0.16).

**Figure 5:**
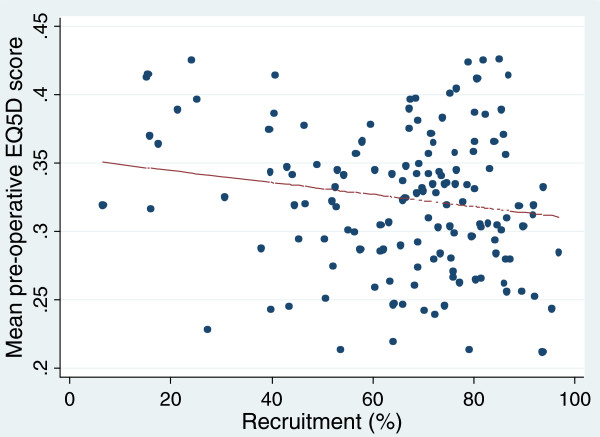
Association between providers’ recruitment rates and mean EQ-5D index score (correlation - 0.18).

For all procedures there was lower recruitment among non-whites (OR 1.25-1.67) and among the most deprived quintile than in the other four quintiles (OR 1.11-1.23). There was no significant association between recruitment rates and patient pre-operative health status (disease-specific PROM score) or quality of life (EQ-5D score). For hip replacement (Figures 4 and 5) the correlations with Oxford Hip Score was -0.16 (p = 0.09) and for EQ-5D -0.18 (p = 0.06). Similar associations were seen with the other three operations (Additional file 2). The slight impact of a 10% higher recruitment rate on the PROM scores is shown in Table 3.

**Table 3:** Association between provider recruitment rate and pre-operative PROM scores

	**Mean score associated with a 10% higher recruitment rate**			
	**EQ5D index score**		**Disease specific score**	
	**Coefficient (95% CI)**	**p-value**	**Coefficient (95% CI)**	**p-value**
Hip replacement	-0.004 (-0.009, 0.000)	0.063	-0.12 (-0.26, 0.02)	0.089
Knee replacement	-0.003 (-0.008, 0.002)	0.301	-0.09 (-0.23, 0.05)	0.216
Groin hernia repair	-0.002 (-0.005, 0.001)	0.113	-	-
Varicose vein surgery	0.002 (0.000, 0.005)	0.099	-0.15 (-0.28, -0.02)	0.020

Lower scores indicate greater severity for all measures except the condition specific score for varicose vein surgery.

## Discussion

### Main findings

Adopting a pragmatic approach, it is possible to establish a database of linked data (PROMs and HES) that encompasses 76-86% of patients. The process of doing so revealed several reasons why estimates of providers’ recruitment rates based on a simple comparison of aggregated counts of pre-operative questionnaires and hospital episodes rather than refined methods based on linked data will be inaccurate, the impact varying by operation: inclusion of ineligible operations (1.9% - 7.0%); attribution to wrong provider (2.4% - 4.9%); operation cancelled (1.9% - 3.6%).

Estimated national recruitment rates based on linked data and excluding ineligible questionnaires were lower: hip replacement was 73% (rather than 78%), knee replacement 72% (81%), hernia repair 52% (55%) and VV surgery 43% (45%). A more conservative approach in which those of uncertain eligibility are excluded inevitably reduces the estimates even further.

Use of these linked data eliminated providers with estimates of over 100% recruitment though the wide range of rates persisted (eg 6% - 96% for hip replacement for NHS Trusts). The new estimates showed that while over 80% recruitment is feasible for providers, this was achieved by only a quarter of them for hip and knee surgery and by only 2-4% for hernia repair and VV surgery. Many providers achieved less than 50% recruitment: a fifth of hip and knee surgery providers and about 60% of those providing hernia and VV surgery. These differences reflect the way providers have organised the recruitment of patients. Anecdotal evidence suggests that in the providers achieving the highest rates, one member of staff (usually a senior nurse) has taken personal responsibility for ensuring that patients are invited to participate.

While the principal determinant of recruitment rates was the provider, some patients’ socio-demographic characteristics had an influence. Non-white patients (Odds ratio 1.25-1.67) and those from the most deprived socio-economic group (OR 1.11-1.23) were less likely to be recruited in all four operations. This was also true for those aged over 75 years (OR 1.28-1.79) for three operations (not for knee replacement). Sex had little association with recruitment rate. There was no significant association between providers’ recruitment rates and the level of patients’ clinical need.

### Limitations

The method of establishing linked data required two assumptions. First, the best estimates of national recruitment rates assume that those of uncertain eligibility (because they could not be linked) were indeed eligible. This is justified as most linked patients were eligible (eg 92.4% of hip replacements).

Second, recruitment rates do not allow for patients who have more than one eligible hospital episode but only complete one pre-operative questionnaire. Providers have discretion in whether they offer another questionnaire to a patient having a second eligible operation. However, the number of such patients is so small as to have little impact on the recruitment rate.

## Conclusions

These findings have two implications when using PROMs routinely to compare providers: how to calculate recruitment rates and the impact of non-recruitment on comparisons.

First, existing simple methods for estimating recruitment rates give inaccurate estimates. We have demonstrated how linked data can be used to establish consistent inclusion criteria and refine the estimation of recruitment rates for routine use of PROMs. The same principles and methods would also be applicable when estimating recruitment rates for other national clinical databases that use routine hospital administrative data to provide estimates of denominators.

Second, lower recruitment rates among non-whites, those over 75 years of age and those most deprived may reflect either that these patients are less likely to be asked to complete a questionnaire, less likely to agree to participate, or there is less success in linking their PROMs data to the routine administrative data. This will clearly bias comparisons of the pre-operative case-mix of providers. But will it bias comparisons of providers’ outcomes? If non-recruitment is random then it may not be a problem, even at low levels of recruitment. And even if non-recruitment is largely systematic it may pose no problem if the characteristics in question (confounders) are known, measured and adjusted for. The problem arises if there are other confounders (eg body mass index) that are not accounted for and the distribution of such factors differs between providers (which is possible). Therefore, given that it is impossible to be sure that all important confounders have been accounted for, it is safest to assume that comparisons of providers’ outcomes may be biased by non-recruitment. Clearly the higher the recruitment rate, the less likely the outcomes will be biased. It is therefore important to strive for high recruitment rates to enable pre-operative comparisons to be made.

## Competing interests

The authors declare that they have no competing interests.

## Authors’ contributions

The study was conceived by NB and JvdM; all authors contributed to the methodological design; AH analysed the data; all authors contributed to interpretation of the data; AH and NB wrote the first draft; all authors contributed to redrafting. All authors read and approved the final manuscript.

## Pre-publication history

The pre-publication history for this paper can be accessed here:

http://www.biomedcentral.com/1472-6963/14/66/prepub

## Supplementary Material

Additional file 1 Distribution of NHS Trust recruitment rates for knee replacement, hernia repair and VV surgery before and revision. Click here for file

Additional file 2 Association between providers' recruitment rates and mean pre-operative PROM scores for knee replacement, hernia repair and VV surgery before and revision. Click here for file
