# Supplementary material for: Estimating recruitment rates for routine use of patient reported outcome measures and the impact on provider comparisons
Source: BMC Health Serv Res. 2014 Feb 11;14:66. doi: 10.1186/1472-6963-14-66 (PMC3923248; doi:10.1186/1472-6963-14-66)
Supplement: Additional file 1 — Distribution of NHS Trust recruitment rates for knee replacement, hernia repair and VV surgery before and revision. [file 1472-6963-14-66-S1.docx]

**Additional file 1**

**Knee replacements: original participation rates for NHS Trusts (n=144)**

**Knee replacements: revised participation rates for NHS Trusts (n=144)**

**Knee replacements: revised participation rates for NHS Trusts (n=144)**

**Groin hernia repair: original participation rates for NHS Trusts (n=146)**

**Groin hernia repair: revised participation rates for NHS Trusts (n=146)**

* ordered by original participation

**Groin hernia repair: revised participation rates for NHS Trusts (n=146)**

**VV surgery: original participation rates for NHS Trusts (n=123)**

**VV surgery: revised participation rates for NHS Trusts (n=123)**

* ordered by original participation

**VV surgery: revised participation rates for NHS Trusts (n=123)**
