# Supplementary material for: Estimating recruitment rates for routine use of patient reported outcome measures and the impact on provider comparisons
Source: BMC Health Serv Res. 2014 Feb 11;14:66. doi: 10.1186/1472-6963-14-66 (PMC3923248; doi:10.1186/1472-6963-14-66)
Supplement: Additional file 2 — Association between providers' recruitment rates and mean pre-operative PROM scores for knee replacement, hernia repair and VV surgery before and revision. [file 1472-6963-14-66-S2.docx]

**Additional file 2**

**Knee replacement**

**Association between providers’ recruitment rates and mean Oxford Knee Score**

**Association between providers’ recruitment rates and mean EQ-5D**

**VV surgery**

**Association between providers’ recruitment rates and mean AVVQ score**

**Association between providers’ recruitment rates and mean EQ-5D**

**Groin hernia repair**

**Association between providers’ recruitment rates and mean EQ-5D**
